# Hybrid fuzzy logic approach for enhanced MPPT control in PV systems

**DOI:** 10.1038/s41598-025-03154-w

**Published:** 2025-06-02

**Authors:** Mustapha Melhaoui, Mohammed Rhiat, Mohammed Oukili, Ilias Atmane, Kamal Hirech, Badre Bossoufi, Mishari Metab Almalki, Thamer A. H. Alghamdi, Mohammed Alenezi

**Affiliations:** 1 LSEEET Laboratory, Department of Applied Physics, Faculty of Science and Technology, Cadi Ayyad University of Marrakech, Marrackech, Morocco; 2 PESIP Team - LGEM Laboratory, Mohammed First University, Oujda, Morocco; 3 Higher School of Education and Training, Mohammed First University, Oujda, Morocco; 4 Laboratory of Engineering Modeling and Systems Analysis, SMBA University, Fez, Morocco; 5 Department of Electrical Engineering, Faculty of Engineering, Al-Baha University, 65779-7738 Alaqiq, Saudi Arabia; 6 Wolfson Centre for Magnetics, School of Engineering, Cardiff University, Cardiff, CF24 3AA UK; 7 Electrical Engineering Department, Faculty of Engineering, Al-Baha University, 65779 Al-Baha, Saudi Arabia

**Keywords:** Photovoltaic (PV) system, Stand-alone photovoltaic systems, Maximum power point tracking (MPPT), Fuzzy logic control (FLC), DC/DC boost converter, Load change, Electrical and electronic engineering, Renewable energy

## Abstract

This paper provides an in-depth analysis of photovoltaic (PV) system control within the MATLAB/Simulink environment, focusing on optimizing Maximum Power Point Tracking (MPPT) algorithms for enhanced efficiency under dynamic conditions. While conventional algorithms are widely used, their performance is limited under fluctuating conditions. To address this, we propose a novel hybrid approach combining Incremental Conductance with Fuzzy Logic Control (FLC), utilizing two innovative input variables: the sum of Conductance and Incremental Conductance (SInC) and its rate of change (CSI). The performance of the proposed algorithm, in comparison to other hybrid FLC methods, is evaluated through simulations using a boost converter under dynamic conditions, including abrupt irradiance changes and load variations. The results demonstrate that the proposed hybrid algorithm achieves superior performance, with an average MPPT efficiency of 97.7%, a convergence time of 53.5 ms, and an RMS of 97.8%, outperforming both conventional and other hybrid techniques. This work advances PV system control by providing a robust and adaptive solution for maximizing power extraction under diverse operating conditions.

## Introduction

The mounting global demand for energy, fueled by industrial expansion and increased consumption, has intensified the need for improved energy efficiency and the adoption of sustainable, renewable energy sources. The depletion of conventional energy reserves, coupled with environmental concerns and geopolitical challenges, has driven up energy costs, underscoring the urgency of transitioning to renewables such as solar and wind power. However, this transition requires not only the deployment of renewable energy systems but also the efficient extraction and delivery of power at competitive costs^1–4^.

Photovoltaic (PV) systems, while environmentally beneficial, face significant challenges that limit their widespread adoption. Industrial PV panels typically suffer from low energy conversion efficiencies (below 20%). Changes in sunlight intensity, ambient temperature, and even partial shading can drastically impact their electrical output. Additionally, the natural degradation of PV panels over time, leading to reduced energy production, further contributes to suboptimal performance, particularly in large-scale PV farms^5^. These combined factors often lead to reduced efficiency and reliability, significantly limiting the overall effectiveness of PV systems^6,7^.

A critical challenge in PV systems lies in their nonlinear electrical characteristics. The Maximum Power Point (MPP) represents the optimal operating condition where the product of voltage ($V_{mpp}$) and current ($I_{mpp}$) is maximized, leading to the highest power output ($P_{mpp}$). Achieving MPP operation requires precise impedance matching, where the load resistance equals the panel’s internal resistance ($R_{mpp} = V_{mpp} /I_{mpp}$). However, load resistance inevitably fluctuates with varying demands, causing the operating point to deviate from the MPP and resulting in significant power losses.

To address this challenge and ensure efficient power transfer, DC/DC power converters (e.g., Boost, Buck, SEPIC) are used, acting as an impedance transformer^8,9^. These converters use power switches controlled by a Pulse Width Modulation (PWM) signal with a fixed frequency and variable duty cycle. Maximum Power Point Tracking (MPPT) algorithms are vital for ensuring optimal power extraction from photovoltaic systems by accurately controlling the duty cycle.

Diverse studies have explored various MPPT techniques to increase the efficiency and robustness of photovoltaic PV systems under dynamic operating scenarios. Conventional algorithms such as Perturb and Observe (P&O)^9,10^, Hill Climbing^11,12^ and Incremental Conductance (InC)^13–15^ are widely used due to their simplicity. However, these methods rely on fixed step sizes for duty cycle adjustments, leading to compromise between tracking speed and accuracy near the MPP. While these methods perform reasonably well under stable conditions, their effectiveness decrease under changing environmental conditions or abrupt load variations, resulting in power losses and reduced efficiency^16–18^. Recent research highlights the limitations of traditional MPPT techniques under partial under fluctuating sun irradiance, under partial shading conditions and load variations, emphasizing the need for adaptive or intelligent MPPT strategies capable of maintaining optimal performance across diverse operating scenarios^19–21^.

Recent advancements in intelligent MPPT techniques, including Fuzzy Logic Control (FLC) and neural networks^22–30^, have demonstrated significant potential in overcoming these limitations. However, many of these approaches either lack adaptability under extreme conditions or require complex computational resources, limiting their practical implementation. Unlike traditional nonlinear controllers, fuzzy logic controllers offer the advantage of dynamically adapting the duty cycle step size based on expert knowledge, even in the absence of a precise mathematical model. This feature enables fuzzy-based algorithms to respond swiftly to changing operation conditions. The performance and design of fuzzy MPPT algorithms are critically influenced by the choice of input and output variables. While the variation in duty cycle ($\Delta D$) is commonly chosen as the output variable, various input variables have been explored in the literature, depending on the approach used to track the MPP. Some authors selected P–V slope ($\Delta P/\Delta V$) and the variation of power ($\Delta P$) as input variables^31^. In other study, the chosen inputs were P–V slope ($\Delta P/\Delta V$) and changes of this slope^30–34^, while other works opted for PV variations in power and voltage ($\Delta P$ and $\Delta V$) or PV variations in power and current ($\Delta P$ and $\Delta I$)^25^.

This research centers on the development and assessment of advanced MPPT control strategies, with a specific focus on fuzzy logic-based MPPT control (Hybrid FLC) approaches. For each algorithm, we analyzed theirs inputs under various operating conditions and established the corresponding membership functions and FLC rules to ensure optimal system performance. We conducted a comprehensive investigation of various Hybrid FLC methods and compared them with conventional techniques.

Furthermore, we propose a novel hybrid approach that integrates Incremental Conductance (InC) MPPT with fuzzy logic control. This method leverages two innovative input variables: the sum of Conductance and Incremental Conductance (SInC) and its rate of change (CSI), to enhance system performance. Through extensive simulations in MATLAB/Simulink, we evaluate the tracking speed, accuracy, and stability of these algorithms around the MPP using key performance metrics such as RMS, RMSE, and efficiency. The evaluation is conducted under extreme operating conditions, including abrupt irradiance changes and sudden load variations, to rigorously test the robustness and adaptability of each algorithm^35–38^.

This work contributes to the ongoing efforts to improve the efficiency and adaptability of PV systems, offering a robust solution for maximizing power extraction under diverse and dynamic operating conditions with a lower cost. By combining the strengths of Incremental Conductance and fuzzy logic control, our proposed approach addresses the limitations of traditional MPPT methods, providing a significant advancement in the field of renewable energy systems.

## PV System description

**Fig. 1:**
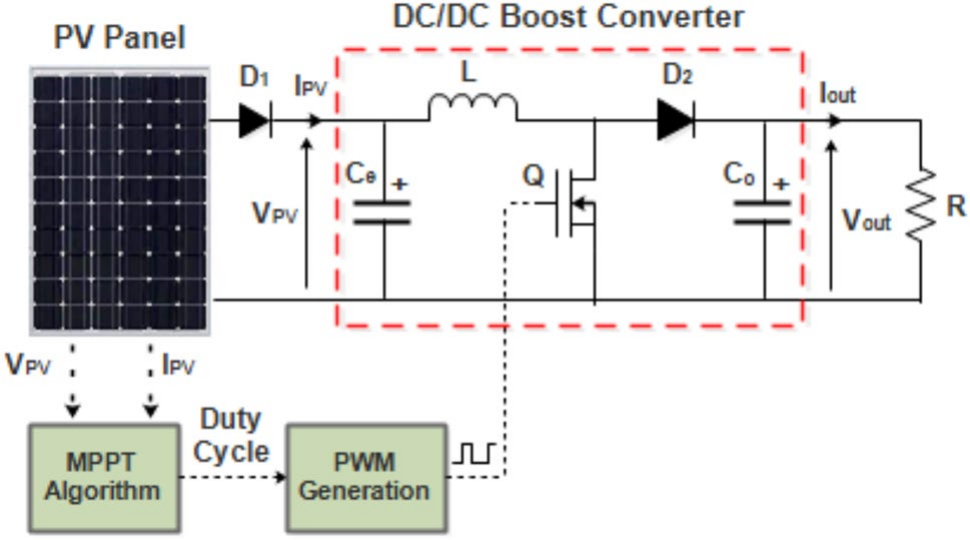
Schematic diagram of PV system.

The complete PV system consist of PV panel, a DC-DC converter regulated by a 10 kHz PWM signal, and a 50 Ω resistive load. The system schematic is depicted in Fig. 1.

**Fig. 2:**
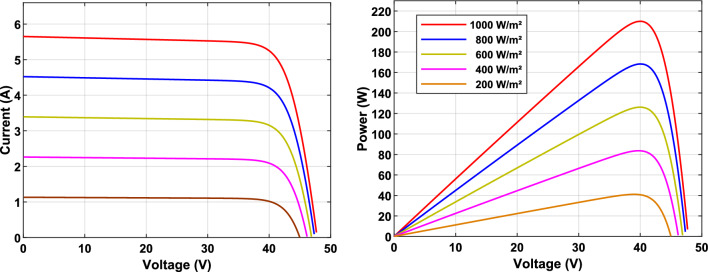
I–V and P–V curves of PV array under different illumination conditions.

A SunPower SPR-210 PV panel was employed for this study. An increase in irradiance results in higher PV power output, whereas an increase in temperature conversely decrease its performance. Figure 2 illustrates the P–V and I–V characteristics under various irradiance conditions. Detailed electrical specifications at STC of the 210W SunPower PV module are presented in Table 1.

**Table 1:** Electrical characteristic of PV panel at STC (SunPower SPR-210).

Open circuit voltage Voc (V)	47.8
Short circuit current Isc (A)	5.65
Voltage at maximum power point Vmp (V)	40
Current at maximum power point Imp (A)	5.25
Maximum power (W)	210
Temperature coefficient of Voc (%/deg.C)	− 0.2792
Temperature coefficient of Isc (%/deg.C)	0.035894

A DC–DC converter is interposed between the PV panel and the load to independently regulate the PV voltage from load conditions and maintain operation at the MPP, as determined by the MPPT controller. The converter topology, as illustrated in Fig. 1, utilizes a MOSFET as the power switch, controlled via a PWM signal.

Under steady-state conditions, a boost converter sustains a constant output voltage. The relationship between the input and output voltages and currents are described by:

1 $$V_{out} = \frac{1}{1 - D}V_{PV}$$

2 $$I_{out} = \left( {1 - D} \right) \cdot I_{PV}$$

Under optimal operating conditions, where the PV generator is interfaced with the load via a DC–DC boost converter, the following relationship holds true:

3 $$D_{mpp} = 1 - \sqrt {\frac{{R_{mpp} }}{R}} ; R_{mpp} = \frac{{V_{mpp} }}{{I_{mpp} }}$$

DC/DC boost converter parameters were determined under standard test conditions (STC). The PV optimal resistance ($R_{mpp}$) was calculated as 7.62 Ω, with an optimal Duty Cycle ($D_{mpp}$) of 0.61, resulting in an output DC/DC voltage ($V_{out}$) of 102 V and current ($I_{out}$) for 2.04 A.

The inductance value (L) is established according to the required current ripple, as determined by the following equation:

4 $$L \ge \frac{{V_{mpp} \cdot D_{mpp} }}{{f \cdot \Delta I_{L} }}$$

The output capacitance value ($C_{o}$) is determined based on the allowable voltage ripple, calculated using the following equation:

5 $$C_{o} \ge \frac{{V_{out} \cdot D_{mpp} }}{{R \cdot f \cdot \Delta V_{out} }}$$

The input capacitor ($C_{e}$) in a DC-DC boost converter is essential for stabilizing the input voltage and reducing voltage ripple. Its sizing is influenced by several factors, including the inductor (L), the damping factor ($\xi$), and $R_{MPP}$. The relationship between these components can be understood through the transfer function that describes how the duty cycle (D) affects the photovoltaic voltage ($V_{pv}$)^39^:

6 $$\frac{{V_{PV} \left( p \right)}}{D\left( p \right)} = - \frac{{V_{out} }}{{LC_{e} \cdot p^{2} + \frac{L}{{R_{pv} }} \cdot p + 1}}$$

The Eq. (6) present the transfer function that can be compared to the general form of a second-order system. By rearranging the damping factor equation, we obtain the expression for the input capacitor ($C_{e}$) as^40^:

7 $$C_{e} = \frac{1}{{4 \cdot \left( {R_{MPP} } \right)^{2} }} \times \frac{L}{{\xi^{2} }}$$

For 4% current ripple and 0.5% voltage ripple respectively, we can calculate $L \approx 12$ mH and $C_{s} \approx 250$ µF. To achieve a fast response time with an acceptable overshoot of 4.6%, we selected a damping factor of $\xi = 0.7$. Substituting the values ($R_{pV} = R_{mpp}$) into the equation, we calculated: $C_{e} = 131,6$ µF. For practical implementation, we chose a standard capacitor value of: $C_{e} = 150$ µF.

## Conventional MPPT control

The nonlinear behavior of PV systems causes variations in power output, which are affected by fluctuations in cell temperature and solar irradiance. For each operating condition, an optimal point exists, as shown in Fig. 2, where the PV array attains its maximum power output and efficiency.

This study explores various MPPT algorithms, including a hybrid approach combining Incremental Conductance and fuzzy control. These algorithms are compared to assess their performance in optimizing PV system power output.

**Fig. 3:**
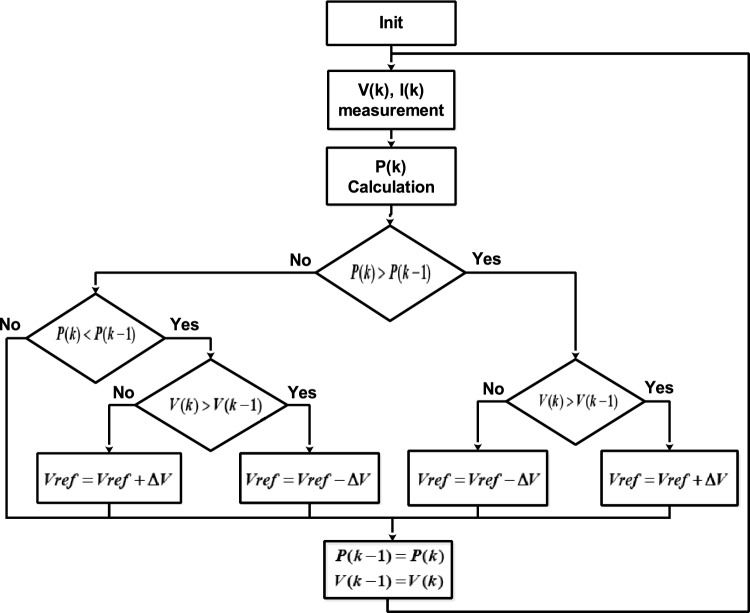
P&O MPPT algorithm.

*The Perturb and Observe (P&O) method* is an intuitive and effective MPPT technique that involves making small perturbations to the system and monitoring the resulting changes in power output to determine the subsequent control action. If the PV power increases ($\Delta P/\Delta V > 0$), the PV reference voltage ($V_{ref}$) is incremented; otherwise, it is decremented (Fig. 3).

**Fig. 4:**
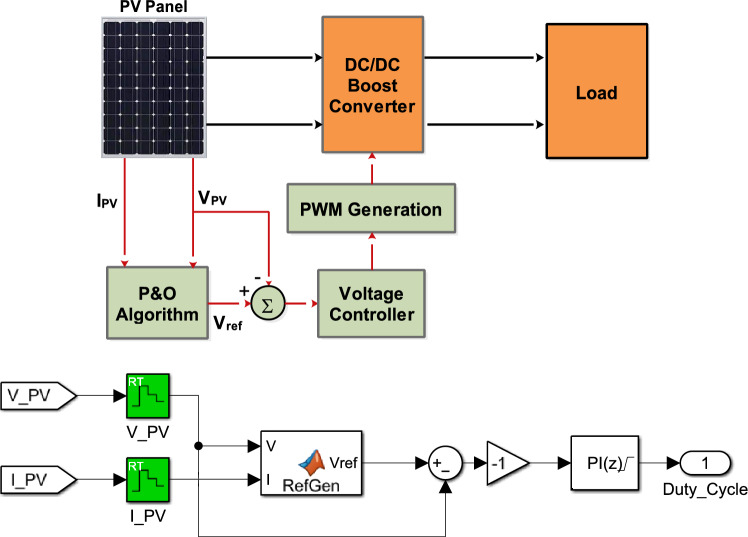
MPPT schematic diagram.

The MPPT controller’s schematic and Simulink implementation are shown in Fig. 4. To ensure proper control direction in the inverter-boost converter system, an inverter block was added before the PI controller, as the system with the boost converter operates inversely^40^.

*The Incremental Conductance (InC)* algorithm offers a more advanced approach to MPPT compared to the P&O method^28^. By evaluating both the instantaneous conductance and the incremental conductance of the PV panel, this algorithm achieves more accurate and efficient MPP tracking. The optimal operation occurs when the derivative of the PV power with respect to the PV voltage is zero, defined as:

8 $$\frac{{dP_{PV} }}{{dV_{PV} }} = 0 \Rightarrow I_{PV} + V_{PV} \cdot \frac{{dI_{PV} }}{{dV_{PV} }} = 0 \Rightarrow \frac{{I_{PV} }}{{V_{PV} }} + \frac{{dI_{PV} }}{{dV_{PV} }} = 0$$

The P–V slope is zero at the MPP, positive to the left of the MPP, and negative to the right of the MPP. In InC algorithm, this translates to:

- ${\text{InC}} = - { }C_{PV} ,$ the operating point is at the MPP;
- ${\text{InC}} > { } - { }C_{PV} ,$ the operating point is to the left of the MPP;
- ${\text{InC}} < { } - { }C_{PV} ,$ the operating point is to the right of the MPP;

where $C_{PV} = { }\frac{{I_{PV} }}{{V_{PV} }}$ is the instantaneous Conductance, and ${\text{InC}}$ is its rate of change, also known as the Incremental Conductance.

**Fig. 5:**
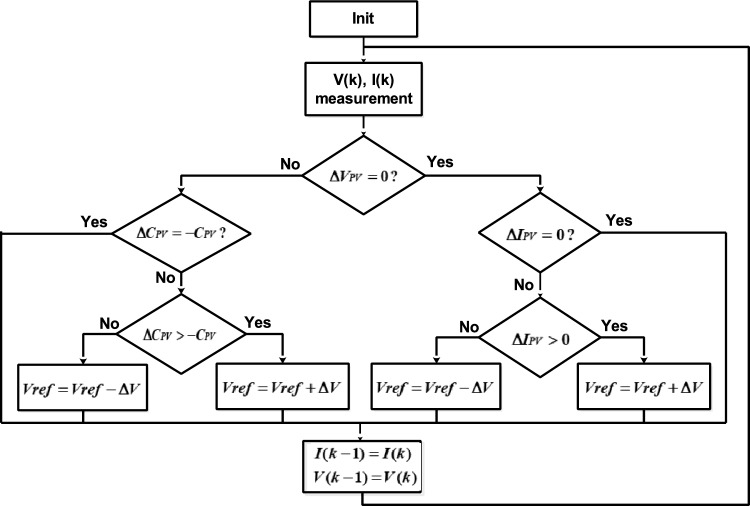
InC MPPT algorithm.

The InC algorithm, shown in Fig. 5, employs $C_{PV}$ and $\Delta C_{PV}$ to determine the control action. The algorithm’s output is the reference voltage:$V_{ref}$. If operating point is located to the right of the MPP, $V_{ref}$ is decreased to shift the operating point leftward. Conversely, if the operating point is in the left side, $V_{ref }$ is increased. A PI controller is employed to regulate the photovoltaic voltage ($V_{PV}$) to match a reference voltage ($V_{ref }$) by adjusting the duty cycle of the PWM sign.

## Fuzzy logic controller

Traditional control methods often struggle to achieve optimal performance in complex systems with nonlinearities or imprecise data. In such scenarios, fuzzy logic offers a valuable alternative by incorporating human expertise and domain knowledge into controller design. This approach uses a rule-based system with "If–Then" guidelines, where variables are expressed as fuzzy sets with varying degrees of membership rather than crisp values. For the DC/DC converter, a Fuzzy Logic Controller (FLC) employing the Mamdani inference technique is utilized^22–24^. Following the fuzzy reasoning process, a defuzzification stage translates the resulting fuzzy output into a crisp control signal.

**Fig. 6:**
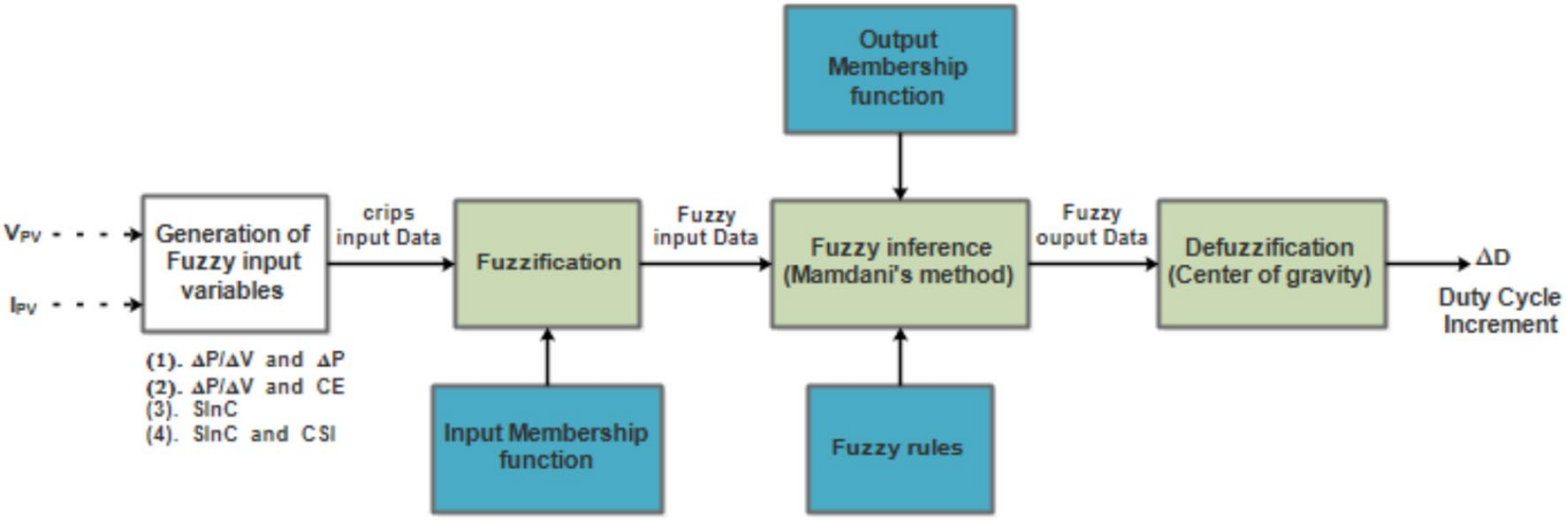
Fuzzy MPPT structure.

Fuzzy MPPT controllers use real-time voltage and current measurements from the PV panel as input variables to regulate the PWM signal, maximizing PV power output. As shown in Fig. 6, the FLC algorithm’s structure revolves around three main components^40^:

- *Fuzzification* This stage converts the crisp input values into fuzzy values using linguistic variables. These linguistic variables facilitate the incorporation of human expertise and domain knowledge into the control strategy.
- *Knowledge Base* This component consists of a set of IF–Then rules, where the rule base is established using linguistic functions to define the relationship between fuzzy input variables and the desired output
- *Defuzzification* This process transforms fuzzy values back into crisp, precise values. Various defuzzification methods exist, such as the centroid method, to achieve this conversion^40,41^.

The FLC employs Mamdani’s fuzzy inference system to determine the optimal increment in duty cycle. The output, representing the duty cycle increment ($\Delta D$), is calculated using the center of gravity method for defuzzification, as follows:

9 $$\Delta D = \frac{{\mathop \sum \nolimits_{j = 1}^{n} \Delta D_{j} \cdot \mu \left( {\Delta D_{j} } \right)}}{{\mathop \sum \nolimits_{j = 1}^{n} \mu \left( {\Delta D_{j} } \right)}}$$

The performance of fuzzy MPPT controllers is significantly influenced by the choice of fuzzy input variables. This study investigates and compares the effectiveness of FLC-MPPT controllers utilizing various combinations of input variables.

Different fuzzy MPPT control methods are discussed in this work, all sharing a common variable output: the duty cycle increment (Fig. 6).

Effective fuzzification requires careful selection of membership function intervals for each input of the fuzzy logic controller to ensure optimal system control. To determine these intervals, we systematically analyzed input variations across the PV system’s operating range, from open circuit to short circuit. This analysis enables a detailed study of the system’s behavior, allowing us to accurately define the necessary membership functions and rigorously formulate fuzzy inference rules. The resulting control strategy aims to optimize MPPT speed, increase tracking accuracy, and enhance system robustness under diverse operating conditions^40^.

**Fig. 7:**
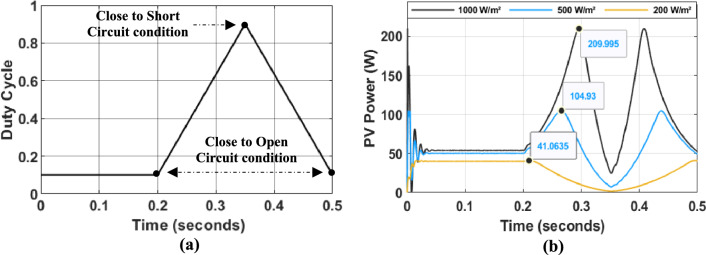
PV System Characteristics. (**a**) Duty cycle variation for system dynamic analysis. (**b**) PV panel characteristics under different illumination conditions.

To evaluate the dynamic performance of the PV system and compare different control strategies, a standardized operating profile was applied. This profile, depicted in Fig. 7, consists of three distinct phases:

- Phase 1 (0–200 ms): System stabilization with a constant duty cycle of 0.1.
- Phase 2 (200–350 ms): Linear ramp of the duty cycle from 0.1 to 0.9.
- Phase 3 (350–500 ms): Linear reduction of the duty cycle from 0.9 to 0.1.

Experiments were conducted under varying illumination conditions (1000 W/m^2^, 500 W/m^2^, and 200 W/m^2^) while maintaining a constant temperature of 25 °C. The temporal evolution of photovoltaic panel power ($P_{PV}$) under these conditions is illustrated in Fig. 7b.

### First Hybrid P&O-FLC algorithm using $\Delta P/\Delta V$ and $\Delta P$ as input variables

The first proposed hybrid MPPT algorithm integrates *the Perturb and Observe (P&O) method with FLC*. The FLC utilizes the P–V curve slope ($\Delta P/\Delta V$) and the change in power ($\Delta P$) as input variables.

**Fig. 8:**
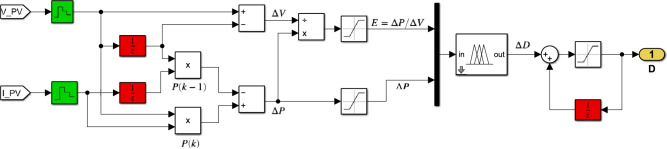
Simulink Circuit Diagram of the Hybrid P&O-FLC MPPT Controller using $\Delta P/\Delta V$ and $\Delta P$ as input variables.

Figure 8 illustrates the first MPPT block system in MATLAB/SIMULINK employing a hybrid P&O-FLC algorithm. This algorithm utilizes the P–V curve slope ($\Delta P/\Delta V$) and power variation ($\Delta P$) as input variables.

**Fig. 9:**
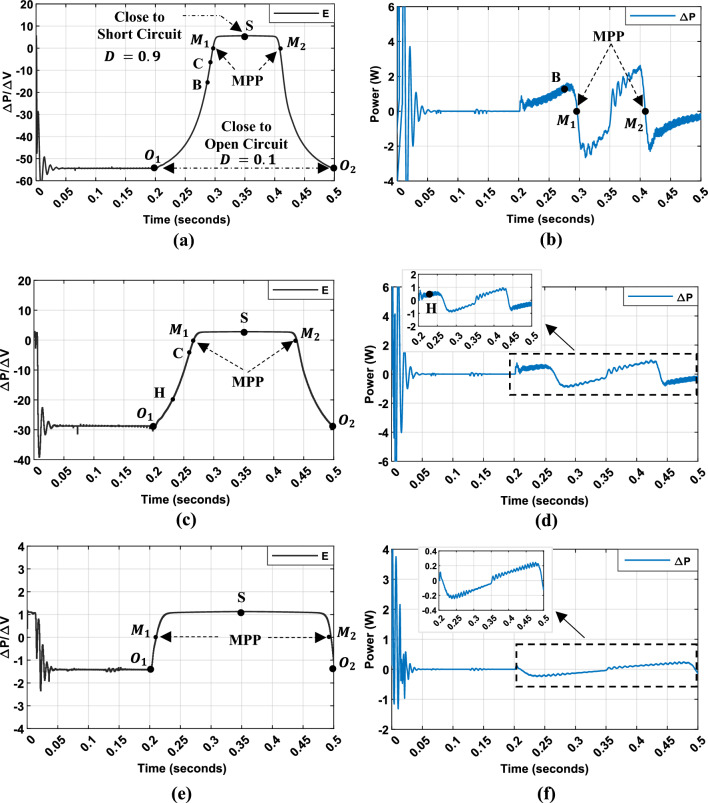
Inputs variable (P–V curve slope $E = \Delta P/\Delta V$ and PV power variation,$\Delta P$) used for Hybrid P&O-FLC under different illumination conditions: (**a**–**b**): under $1000$ W/m^2^ (**c**–**d**): under $500$ W/m^2^. (**e**–**f**): under $200$ W/m^2^.

Figure 9a–f, presents the dynamic response of a PV panel to duty cycle variations, as shown in Fig. 7a. The slope ($E = \Delta P/\Delta V$) and power variation ($\Delta P$) are studied for duty cycles ranging from 0.1 and 0.9, under different irradiance levels (200 W/m^2^, 500 W/m^2^, 1000 W/m^2^) while maintaining a constant temperature of 25 °C.

To optimize MPPT performance, the fuzzy rule base is segmented into distinct regions defined by P–V curve slope ($E = \Delta P/\Delta V$) and power variation ($\Delta P$) characteristics (Fig. 9a–f). Control strategies are defined as follows:

- Negative P–V slope region ($E < 0$): In this region, the operating point is located to the right of the MPP on P–V characteristic curve. Consequently, an increase of the duty cycle is necessary to shift the operating point towards the MPP. The duty cycle adjustment is based on the distance from the MPP, which is assessed using $E$ and $\Delta P$. This adjustment is designed to improve both the speed and accuracy of the search for the MPP:
  - If $E$ is NB, as shown for example at Point $O_{1}$ and $O_{2}$ in Fig. 9a–c, the operating point is far from the MPP. Therefore, a significant increase in the duty cycle is required for rapid convergence.
  - If $E$ is NM, as shown at Point B and Point H in Fig. 9a–d, proximity to the MPP is established. However, we can further improve our MPPT control by using the secondary input, $\Delta P$:
    - If $\Delta P$ is big (Point B in Fig. 9b), a gradual decrease in the increment value is recommended to prevent overshooting the MPP.
    - Otherwise, a moderate increase in the duty cycle can be implemented since the MPP has not yet been reached (Point H in Fig. 9d).
  - If $E$ is NS, as shown at Point C in Fig. 9a–d, the operating point is near the MPP. In this situation, only a minor adjustment to the duty cycle is necessary. However, if $\Delta P$ is zero, the duty cycle should remain unchanged to prevent overshooting the MPP, especially under low irradiance conditions since the optimal duty cycle is close to $0.1$ (e.g., $D_{mpp} \approx 0.138$ when $Le = 200$ W/m^2^).
- *Zero P–V slope (*$E$ *is ZE)*: the operating point coincides with the MPP, as shown at Point $M_{1}$ and $M_{2}$ in Fig. 9a–f. The duty cycle should remain unchanged. To further improve accuracy, $\Delta P$ can be used:
  - If $\Delta P$ is very small, the MPP has been reached.
  - If $\Delta P$ is large, a slight increment in duty cycle can be performed, depending on the sign of $\Delta P$, to match the MPP.
- *Positive P–V slope region* the operating point is positioned to the left of the MPP. Therefore, the system must decrease the duty cycle value. The same logic, employing Fig. 9, applies for establishing MPPT control laws. However, consider the following:
  - When E is PM, and $\Delta P$ is very small, then a gradual decrease in the increment value is recommended to prevent overshooting the MPP.
  - When E is PM and $\Delta P$ is significant, a moderate decrease in the duty cycle is recommended to improve tracking speed under low and high irradiance conditions.
- However, under low irradiance conditions (Fig. 9e–f), the duty cycle is adjusted more gradually in response to power variation ($\Delta P$). This adjustment is necessary because the slope of the PV curve is significantly smaller compared to its values under high irradiance conditions. As a result, the duty cycle variation is less responsive to the operating point’s distance from the MPP, necessitating a more refined control approach to maintain optimal performance.

The system’s output is the duty cycle value, which is adjusted by the FLC-MPPT controller. Table 2 presents the fuzzy rule base designed using these input variables.

**Table 2:** Fuzzy rule base using P–V Curve Slope and Power variation as input Variables.

Fuzzy rules		$E\left( k \right) = ~\Delta P_{{PV}} /\Delta V_{{PV}}$						
		NB	NM	NS	ZE	PS	PM	PB
$\Delta P_{PV}$	NB	PB	PS	PS	PS	NS	NB	NB
	NM	PB	PM	PS	PS	NS	NM	NB
	NS	PB	PM	PS	ZE	NS	NS	NB
	ZE	PB	PM	ZE	ZE	ZE	NS	NB
	PS	PB	PM	PS	ZE	NS	NS	NB
	PM	PB	PM	PS	NS	NS	NM	NB
	PB	PB	PS	PS	NS	NS	NB	NB

### Second Hybrid P&O-FLC algorithm using $\Delta P/\Delta V$ and $CE$ as input variables

The second proposed hybrid MPPT algorithm integrates also *the Perturb and Observe (P&O) method with fuzzy logic control*. To enhance tracking accuracy, the FLC uses the P–V curve slope ($E = \Delta P/\Delta V$) and the change in this slope ($CE$) as primary input variables. These parameters are calculated as follows:"

10 $$E\left( k \right) = \frac{{\Delta P_{PV} }}{{\Delta V_{PV} }} = \frac{{I_{PV} \left( k \right)V_{PV} \left( k \right) - I_{PV} \left( {k - 1} \right)V_{PV} \left( {k - 1} \right)}}{{V_{PV} \left( k \right) - V_{PV} \left( {k - 1} \right)}}$$

11 $$CE\left( k \right) = E\left( k \right) - E\left( {k - 1} \right)$$

**Fig. 10:**
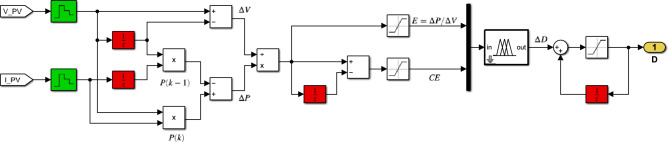
Simulink Circuit Diagram of the Hybrid P&O-FLC MPPT Controller using $\Delta P/\Delta V$ and $CE$ as input variables.

Figure 10 illustrates the second MPPT block system within the MATLAB/Simulink environment, employing a hybrid P&O-FLC algorithm. This algorithm utilizes the P–V curve slope ($\Delta P/\Delta V$) and its rate of change ($CE$) as input variables^40^.

**Fig. 11:**
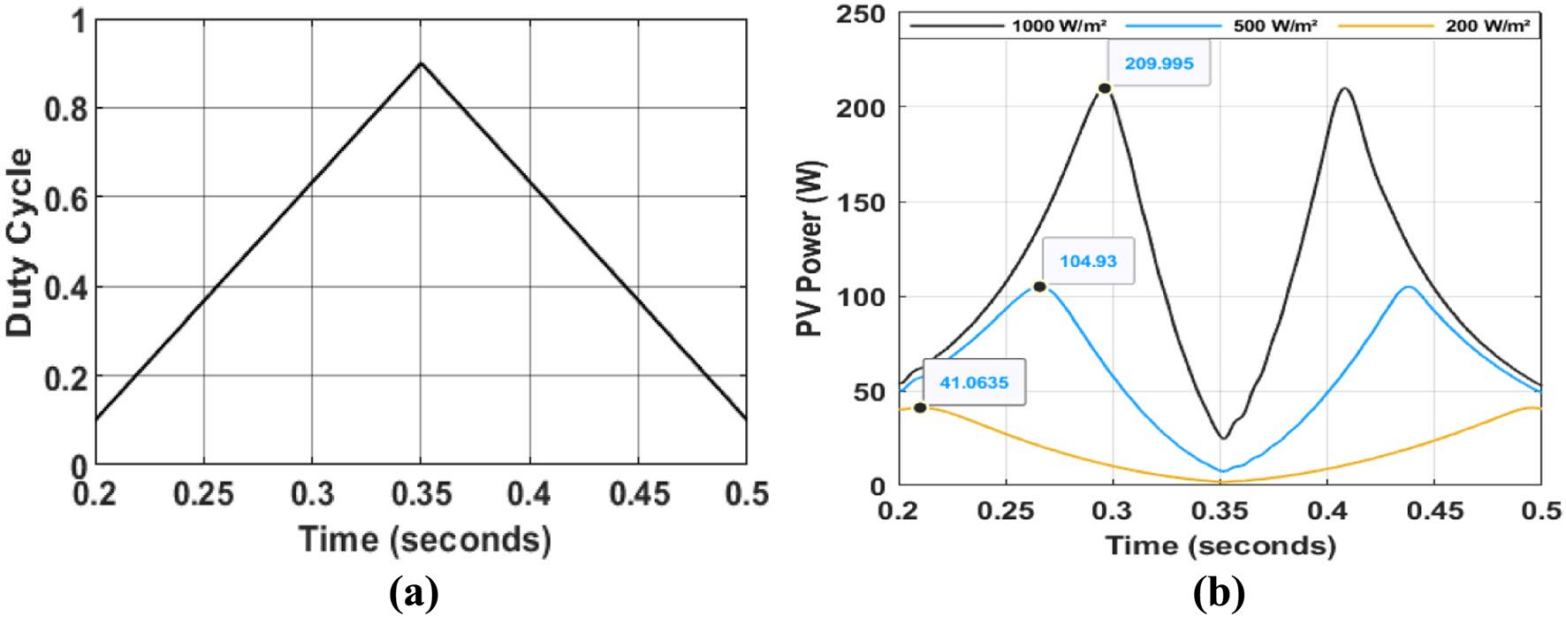
(**a**) Duty cycle variation for system dynamic analysis. (**b**) PV panel characteristics under different illumination conditions.

To evaluate the dynamic performance of the PV system and compare different control strategies, we established a consistent profile focusing on the last two phases (Fig. 11):

- Phase 1 (200–350 ms): Linear ramp of the duty cycle from 0.1 to 0.9.
- Phase 2 (350–500 ms): Linear reduction of the duty cycle from 0.9 to 0.1.

Experiments were conducted under varying illumination conditions (1000 W/m^2^, 500 W/m^2^, and 200 W/m^2^) while maintaining a constant temperature of 25 °C. The same profile was used for the subsequent MPPT algorithms to ensure a fair comparison.

**Fig. 12:**
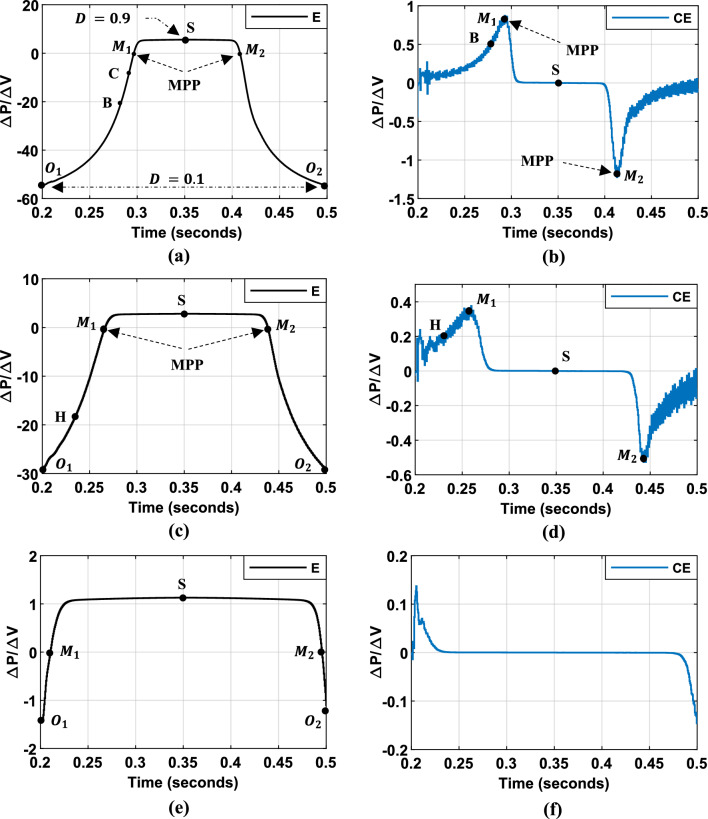
Inputs variable ($E = \Delta P/\Delta V$ and $\user2{ }{\mathbf{CE}}$) for Hybrid P&O-FLC under different illumination conditions: (**a**–**b**): under $1000$ W/m^2^. (**c**–**d**): under $500$ W/m^2^. (**e**–**f**): under $200$ W/m^2^.

Figure 12 illustrates the dynamic response of a PV panel subjected to the varying duty cycle profile depicted in Fig. 11a. The figure analyzes the PV slope ($E=\Delta P/\Delta V$) and the rate of change of slope ($CE$) under different irradiance levels at a constant temperature of 25 °C.

For this algorithm, the fuzzy rule base is segmented based on P–V curve slope ($E = \Delta P/\Delta V$) and its rate of change ($CE$) (Fig. 12):

- In the negative P–V slope region ($E < 0$), the operating point is located to the right of the MPP. A duty cycle increment is applied, with its magnitude determined by the two inputs $E$ and $CE$:
  - If $E$ is NB, as shown at Point $O_{1}$ and $O_{2}$ in Fig. 12a–d, the operating point is far from the MPP. Therefore, a larger duty cycle is applied to accelerate convergence.
  - If $E$ si NM, the operating point is near the MPP and the control will improved by using the secondary input, CE:
    - If $CE$ is large, as shown at Point B in Fig. 12b, it mean that the operating point is near the MPP. To prevent overshooting the MPP and causing system oscillations, the output should be set to PS.
    - Otherwise, as shown at Point H in Fig. 12d, a moderate increase in the duty cycle can be implemented since the MPP has not yet been reached.
  - If $E$ is NS, as shown at Point C in Fig. 12a, a slight increment in the duty cycle is required, since The operating point is very near the MPP. However, if $CE$ is large, the duty increment of duty cycle is set to ZE to prevent overshooting the MPP, especially under low irradiance conditions. This precaution helps avoid excessive adjustments that could destabilize the system.
- *In the Zero P–V slope (*$E$ *is ZE)*, the operating point is at the MPP. The duty cycle should remain unchanged. To further improve accuracy under low irradiance conditions, where both $E$ and $CE$ are very small, the following rule can be added:
  - If CE is very small and not equal to zero, then an increment of duty cycle is required based on its sign.
- *In the Positive P–V slope region*, the operating point is positioned on the left side of the MPP. The system should decrease the duty cycle value using inputs variables: $E$ and $CE$ (Fig. 12) to adjust the duty cycle magnitude. To enhance controller performance under low irradiance conditions, especially when the operating point is distant from the MPP, the following rule can be incorporated:
  - When E is PM, and $CE$ is ZE: A large decrease in the increment value is then applied to reach the PPM faster.
- In cases where the operating point is near to the MPP, $\Delta P$ and $\Delta P$ can approach zero, resulting in large $E$ and $CE$. In these situations, the output can be set to $PS$ or $NS$ as appropriate.

The FLC-MPPT controller’s operation is determined by the fuzzy rule base presented in Table 3, ensuring smooth system operation.

**Table 3:** Fuzzy rule base using P–V Curve Slope and the change in this slope as input variables.

Fuzzy rules		$E\left( k \right) = ~\Delta P_{{PV}} /\Delta V_{{PV}}$						
		NB	NM	NS	ZE	PS	PM	PB
$CE\left( k \right)$	NB	PS	PS	ZE	ZE	ZE	NS	NB
	NM	PB	PS	ZE	PS	ZE	NS	NB
	NS	PB	PM	PS	ZE	NS	NM	NB
	ZE	PB	PM	PS	ZE	NS	NB	NB
	PS	PB	PM	PS	ZE	NS	NM	NB
	PM	PB	PS	ZE	NS	ZE	NS	NB
	PB	PS	PS	ZE	ZE	ZE	NS	NS

### Third Hybrid InC-FLC algorithm using $SCIC$ as input variable

The third hybrid FLC-MPPT method uses *the InC algorithm’s foundation*, which relies on the Eq. 8. This FLC-MPPT method utilizes a single fuzzy input variable, the Sum of Conductance and Increment of Conductance $\left( {SInC = \frac{{I_{PV} }}{{V_{PV} }} + \frac{{dI_{PV} }}{{dV_{PV} }}} \right)$, as the input to the fuzzy logic MPPT controller, to modulate the PWM signal’s duty cycle^40^.

**Fig. 13:**
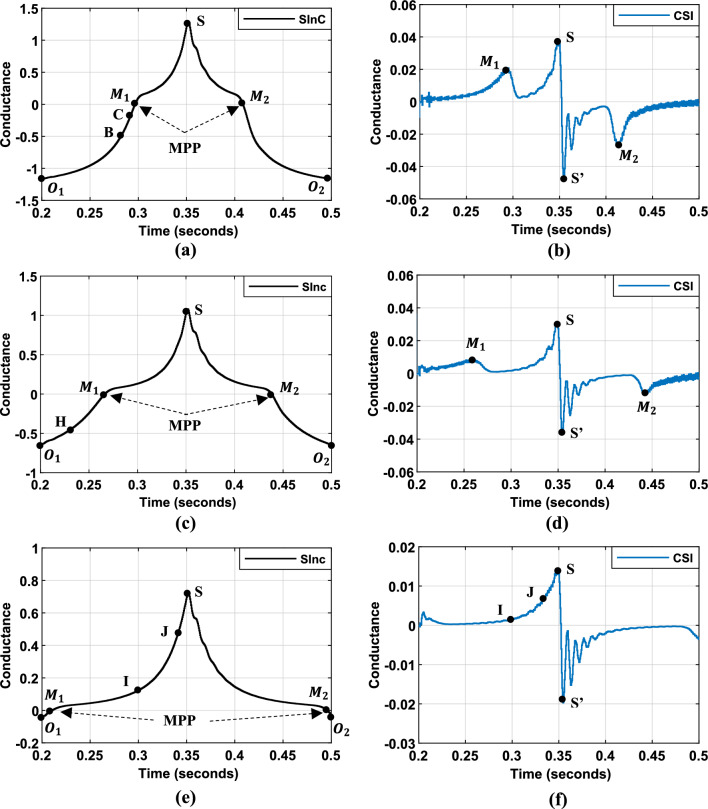
Inputs variables ($SInC$ and its variation ${ }CSI$) for Hybrid InC-FLC under different illumination conditions: (**a**–**b**): under $1000$ W/m^2^. (**c**–**d**): under $500$ W/m^2^. (**e**–**f**): under $200$ W/m^2^.

To evaluate the dynamic performance of the system, the same consistent profile as described in the previous paragraph was employed, as illustrated in Fig. 11. Figure 13 illustrates the evolution of $SInC$ under various irradiance levels and operating points. $SInC$ serves as the sole input for the third MPPT algorithm. Additionally, the figure presents the rate of change of $SInC$ ($CSI$) used in the fourth FLC-MPPT method.

The algorithm employs a single fuzzy input variable ($SInC$) (Fig. 13), with the fuzzy rule base segmented as follows:

- $SInC < 0$: Operating point is right of the MPP. A duty cycle increment is applied, with magnitude determined by Inc value:
  - $SInC$ is NB, as shown at Point $O_{1}$ and $O_{2}$ in Fig. 13a–c: a substantial increment in the duty cycle should be introduced.
    If $SInC$ is NM, as shown at Point B in Fig. 13a and Point H in Fig. 13c: A moderate increase in the duty cycle should be implemented. This adjustment is necessary because the operating point is approaching the MPP and a balanced approach helps fine-tune the system’s performance without overshooting the MPP.
  - If $SInC$ is NS, as shown at Point C in Fig. 13a: a slight duty cycle increment is required to prevent system oscillation.
- $SInC$ is ZE: Operating point at the MPP. The the duty cycle value should be maintained.
- $SInC > 0$: Operating point is left of the MPP. The duty cycle should be decreased based on the value of $SInC$.

**Fig. 14:**
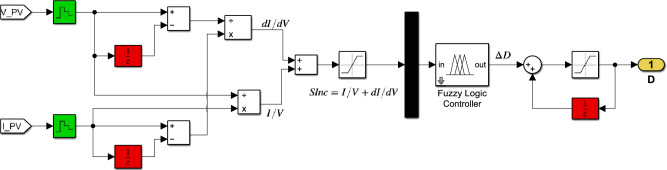
Simulink Circuit Diagram of the Hybrid InC-FLC MPPT Controller using $SInC{ }$ as single variable input.

Figure 14 depicts the Simulink implementation of the hybrid InC-FLC MPPT algorithm. This algorithm employs $SInC$ as the sole input variable. The MPPT controller’s operation is set by the fuzzy rule base presented in Table 4.

**Table 4:** Fuzzy rule base for the InC-FLC MPPT controller, using $SInC$ as single input variable.

Fuzzy Rules	$SInC = \frac{{I_{{PV}} }}{{V_{{PV}} }} + \frac{{dI_{{PV}} }}{{dV_{{PV}} }}$						
	NB	NM	NS	ZE	PS	PM	PB
	PB	PM	PS	ZE	NS	NM	NM

### The proposed Hybrid InC-FLC algorithm using $SInC$ and CSI as input variables

To enhance MPPT tracking speed and accuracy, especially under low irradiance conditions, a second hybrid algorithm incorporating $SInC$ and its rate of change (CSI) as fuzzy inputs is proposed (Fig. 13). The fuzzy rule base is expanded upon the initial approach with the following modifications:

- Negative $SInC$: Duty cycle is increased. Increment magnitude is adjusted based on $SInC$ value. To prevent overshooting the MPP, the duty cycle is maintained when $SInC$ is NS and CSI is large.
- $SInC = 0$: Duty cycle maintained.
- The same reasoning is applied when $SInC$ is positive, except with specific rules to enhance the performance under low irradiance conditions:
  - $SInC$ is PS and CSI is ZE, as shown at Point I in Fig. 13e–f: In this condition, the operating point is moderately distant to the left of the MPP. Therefore, the fuzzy output is set to NM.
  - $SInC$ is PM and CSI is ZE, as shown at Point J in Fig. 13e–f: The operating point is far to the left of the MPP. Therefore, the fuzzy output is set to NB.

These two additional rules enhance MPP tracking under low irradiance conditions. They also allow for fine-tuning all the membership function boundaries, leading to improved overall system performance.

**Fig. 15:**
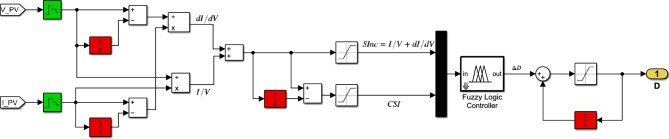
Simulink Circuit Diagram of the Hybrid InC-FLC MPPT Controller using $SInC$ and $CSI$ as input variables.

Figure 15 depicts the Simulink implementation of the hybrid InC-FLC MPPT algorithm. This algorithm uses $SInC$ and its rate of change (CSI) as fuzzy inputs. Table 5 presents the fuzzy rule base designed for this algorithm^40^.

**Table 5:** Fuzzy rule base for the InC-FLC MPPT controller, using $SInC$ and its rate of change (CSI) as input variables.

Fuzzy rules		$SInC = \frac{{I_{{PV}} }}{{V_{{PV}} }} + \frac{{dI_{{PV}} }}{{dV_{{PV}} }}$						
		NB	NM	NS	ZE	PS	PM	PB
$CSI$	NB	PB	PM	ZE	ZE	ZE	NM	NB
	NS	PB	PM	PS	ZE	NS	NM	NB
	ZE	PB	PM	PS	ZE	NM	NB	NB
	PS	PB	PM	PS	ZE	NS	NM	NB
	PB	PB	PM	ZE	ZE	ZE	NM	NB

**Fig. 16:**
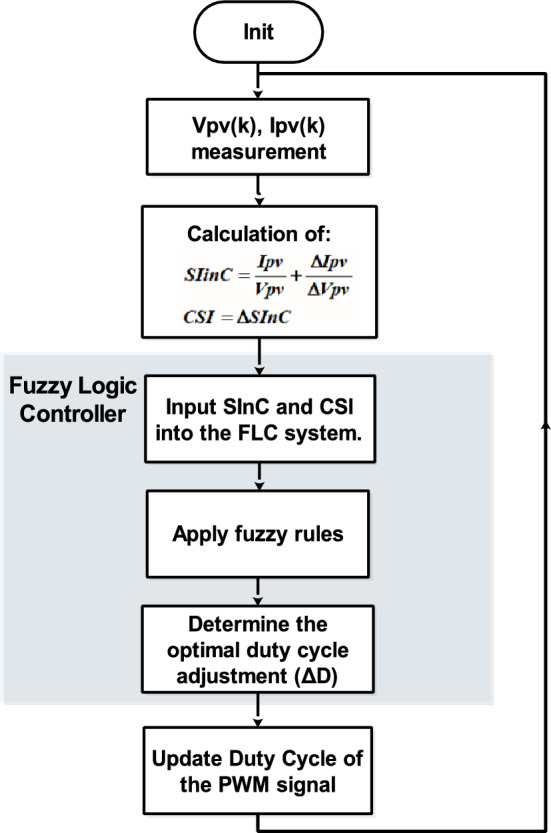
Flowchart of our proposed algorithm.

Figure 16 presents a detailed flowchart of the proposed fuzzy logic-based MPPT control strategy, designed for real-time operation. The process begins with the measurement of PV panel voltage and current, followed by the calculation of the sum of Conductance and Incremental Conductance ($SInC$) and its rate of change ($CSI$). These inputs are fed into the FLC, which applies predefined rules to classify the duty cycle adjustment ($\Delta D$)^40^.

The system continuously updates the duty cycle and controls the DC-DC converter to maintain operation at the MPP. A feedback loop ensures that the system dynamically adapts to changes in irradiance, temperature, and load conditions. If the system is far from the MPP, $\Delta D$ is set to big or medium; if near the MPP, $\Delta D$ is set to small or zero. This continuous adjustment ensures optimal performance under varying operating scenarios.

## Simulation results and discussion

A MATLAB/Simulink simulation of a PV system was developed to assess the dynamic performance of various MPPT techniques. The system comprised a SunPower SPR 210 PV panel, a DC-DC boost converter controlled by a 10 kHz PWM signal, and a 50Ω resistive load (Fig. 1). The simulation was designed to evaluate the effectiveness of the proposed MPPT control strategies under dynamic meteorological conditions and sudden load variations.

Key performance metrics were used to quantify the effectiveness of each algorithm, including:

- *Average MPPT Efficiency* in order to measure the controller’s ability to extract the maximum available power from the PV array expressed as:
  12 $$MPPT Efficiency = \left( {\frac{{\mathop \sum \nolimits_{i = 1}^{N} P_{pv} \left( i \right)}}{{\mathop \sum \nolimits_{i = 1}^{N} P_{opt} \left( i \right) }} } \right) \times 100 \left( \% \right)$$
- *Convergence Time* to quantify the speed at which the MPPT controller reaches the new MPP after a change in operating conditions.
- *Root Mean Square Error (RMSE)* to evaluate the tracking accuracy by quantifying the deviation between the actual and theoretical optimal power. It is defined as:
  13 $$RSME = \sqrt {\frac{1}{N}\mathop \sum \limits_{i = 1}^{N} \left( {P_{pv} \left( i \right) - P_{opt} \left( i \right)} \right)^{2} }$$
- *Normalized RMS Value (RMS%)* with respect to the optimal power in order to assesse the stability of the power output relative to the optimal power. It is calculated as:
  14 $$RMS_{\% } = \frac{{\sqrt {\frac{1}{N}\mathop \sum \nolimits_{i = 1}^{N} P_{pv} \left( i \right)^{2} } }}{{P_{opt} }}$$

### Testing robustness under dynamic irradiance conditions

**Fig. 17:**
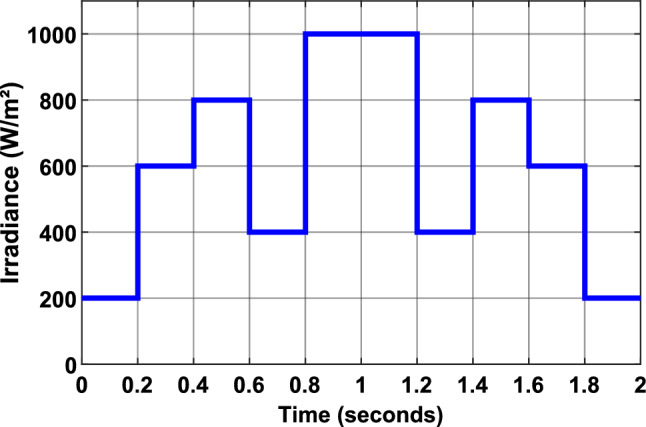
Irradiance profile.

The study was conducted under dynamic conditions, where irradiance levels were subjected to stepwise changes at 0.2-s intervals, with a fixed ambient temperature of 25 °C (Fig. 17). The irradiance profile ranged from 200 to 1000 W/m^2^, simulating real-world variations in solar irradiance. This dynamic profile was designed to rigorously test the ability of the MPPT algorithms to adapt to rapid changes in environmental conditions, which are critical for practical applications.

**Fig. 18:**
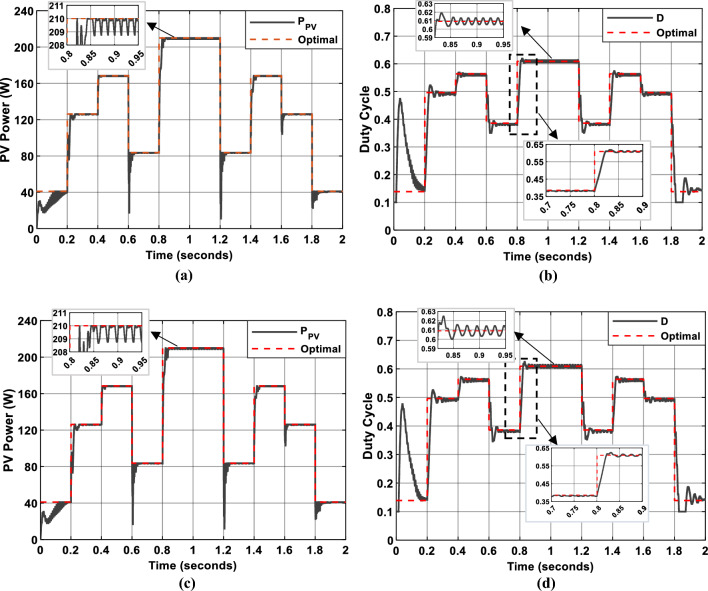
Simulation results of PV System. (**a** and **b**): PV Power and duty cycle using P&O MPPT algorithm. (**c** and **d**): PV Power and duty cycle using Inc MPPT algorithm.

Figure 18 presents shows simulated PV system performance using P&O (Fig. 18a,b) and InC (Fig. 18c,d) MPPT algorithms. For each algorithm, the PV panel power output and duty cycle evolution are depicted (black lines), alongside their optimal values (dotted red lines) over the simulation period, which follows the irradiance profile shown in Fig. 17. The results demonstrate that both the P&O and InC algorithms effectively track the MPP, achieving efficiencies of 95.41% and 95.6%, respectively.

However, both methods exhibit persistent oscillations around the MPP due to continuous duty cycle modulation. These oscillations, while not entirely eliminating the effectiveness of the algorithms, resulted in energy losses and reduced system stability. To address these limitations, a FLC approach can be employed. By dynamically modifying the duty cycle based on the deviation from the estimated MPP, the FLC-based algorithms were able to accelerate convergence and reduce oscillations, thereby improving tracking accuracy and system stability.

**Fig. 19:**
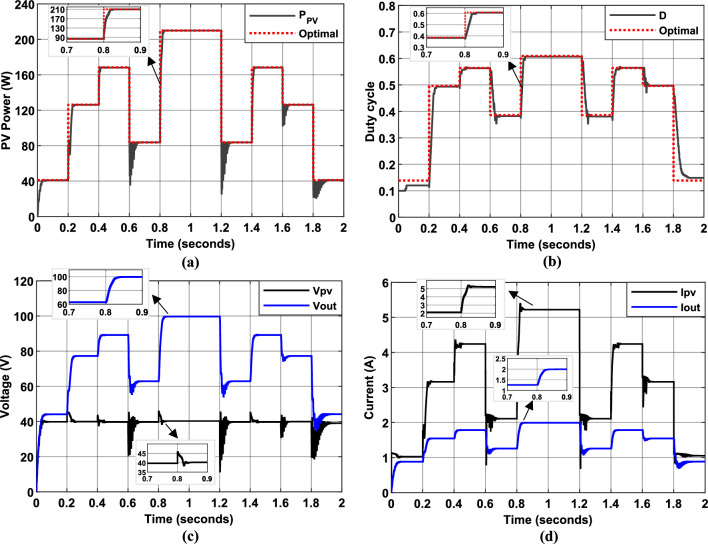
Simulation results of PV System using the first Hybrid MPPT controller. (**a**): PV Power output. (**b**): duty cycle of PWM signal. (**c**): Input and output voltage of DC/DC converter. (**d**): Input and output current of DC/DC converter.

Figure 19 presents the simulation results of the PV system using the first hybrid P&O-FLC MPPT algorithm, which employs $\Delta P/\Delta V$ and $\Delta P$ as input variables. The figure illustrates the PV panel power output, duty cycle evolution, and their corresponding optimal values (represented by dotted red lines) over the simulation period. Additionally, the input and output voltage and current values of the DC/DC converter are displayed (black and blue lines, respectively).

The results demonstrate that the controller successfully tracked the MPPT under fluctuating irradiance conditions, achieving an average MPPT efficiency of 97.5%. During periods of steady irradiance, the electrical quantities remained stable and closely aligned with their optimal values, highlighting the effectiveness of the MPPT control. The duty cycle variation was more substantial when the operating point was distant from the MPP, but it decreased significantly or became negligible as the operating point converged toward the MPP. This adaptive behavior highlights the controller’s ability to dynamically adjust its response based on its proximity to the MPP, ensuring efficient and stable operation under varying environmental conditions.

**Fig. 20:**
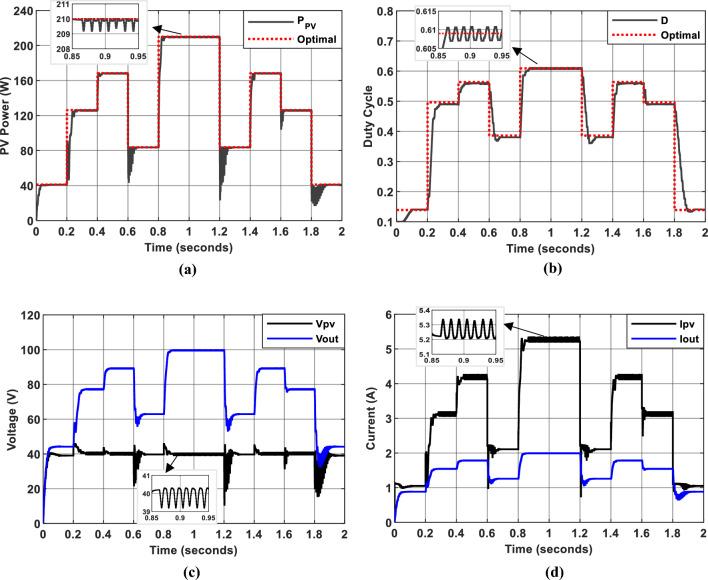
Simulation results of PV System using the second Hybrid MPPT controller. (**a**): PV Power output. (**b**): duty cycle of PWM signal. (**c**): Input and output voltage of DC/DC converter. (**d**): Input and output current of DC/DC converter.

Figure 20 illustrates PV system simulation results using the second hybrid P&O-FLC MPPT algorithm, where the input variables are $\Delta P/\Delta V$ and $CE$. The figure illustrates the PV panel power output, duty cycle evolution, and their corresponding optimal values (represented by dotted red lines) over the simulation period. Additionally, the input and output voltage and current values of the DC/DC converter are displayed (black and blue lines, respectively).

The findings demonstrate the MPPT controller’s effectiveness in precisely tracking the MPP across varying conditions, achieving an average MPPT efficiency of 96.82%. Compared to the previous FLC algorithm (using $\Delta P/\Delta V$ and $\Delta P$ as inputs), the second hybrid P&O-FLC algorithm demonstrates also an improved and tracking speed, particularly near the MPP and under low irradiance conditions. Such a performance is attributed to the inclusion of $CE$, which provides additional information about the rate of change of the P–V curve slope, enabling faster and more precise adjustments to the duty cycle. However, the lower magnitude of $CE$ under high irradiance conditions can introduce minor oscillations in the electrical quantities. To address this, a compromise can be made between reducing oscillations under high irradiance and maintaining high efficiency across all irradiance levels. This trade-off ensures that the algorithm remains robust and effective under a wide range of operating conditions.

**Fig. 21:**
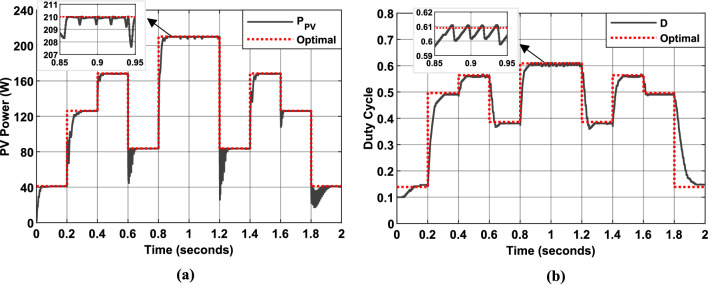
Simulation results of PV System using the third Hybrid MPPT controller. (**a**): PV Power output. (**b**): duty cycle of PWM signal.

Figure 21 illustrates the simulated behavior of the PV system using the third Hybrid InC-FLC algorithm, where $SInC$ (sum of Conductance and Incremental Conductance) serves as the main input variable. The results confirm that this algorithm provides robust MPPT performance under both steady-state and dynamic irradiance conditions, achieving an average MPPT efficiency of 96.6%.

This algorithm is more efficient and easier to implement compared to its predecessors, particularly in terms of precision near the MPP. The $SInC$ value serves as a reliable indicator of the operating point’s proximity to the MPP: a larger $SInC$ value indicates a greater distance from the MPP, while the MPP is reached when $SInC$ equals zero. This contrasts with previous algorithms that relied on the P–V slope ($\Delta P/\Delta V$) as an input variable, which exhibited a limitation: when the operating point is on the right side of the MPP ($\Delta P/\Delta V < 0$), a higher absolute value of P–V slope indicates a greater distance from the MPP. However, when $\Delta P/\Delta V > 0$, there is a point where the P–V slope becomes constant, regardless of the distance from the MPP. This limitation necessitates the use of a second input variable and carefully tuned membership functions and rules to optimize MPPT control.

Despite its advantages, the effectiveness of the InC-FLC algorithm heavily relies on the accuracy of measurement tools, particularly due to the challenges associated with performing accurate division operations. Additionally, as shown in Fig. 21, small oscillations of the electrical quantities around the MPP are observed under high irradiance, and the MPP tracking speed under low irradiance conditions remains suboptimal. To address these limitations, the incorporation of the rate of change of $SInC$ ($CSI$) as an additional input variable greatly improves the algorithm’s precision in identifying and tracking the MPP, especially in challenging operating environments.

**Fig. 22:**
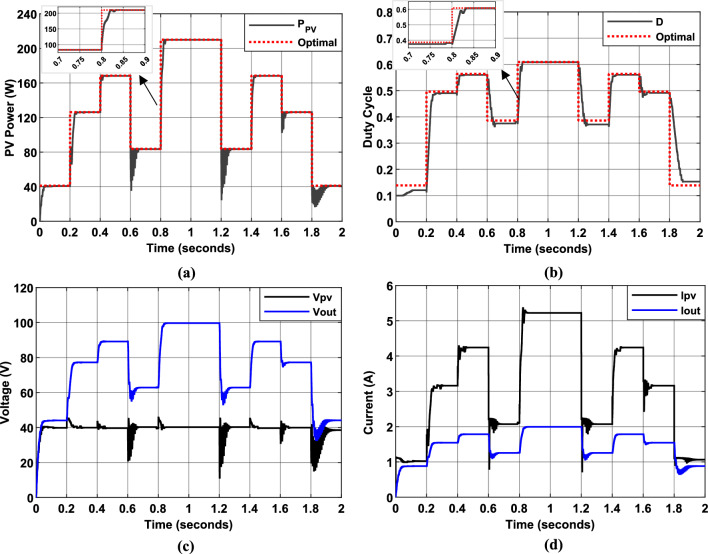
Simulation results of PV System using the fourth Hybrid MPPT controller. (**a**): PV Power output. (**b**): duty cycle of PWM signal. (**c**): Input and output voltage of DC/DC converter. (**d**): Input and output current of DC/DC converter.

Figure 22 illustrates the simulation results of the proposed algorithm. This fourth Hybrid InC-FLC algorithm employs $SInC$ and its rate of change ($CSI$) as fuzzy input variables. The results demonstrate that this algorithm achieves robust MPPT performance, with an average efficiency of 97.7% and rapid, accurate MPP tracking under both high and low irradiance conditions. Notably, the algorithm effectively mitigates oscillations in the electrical quantities, ensuring stable and efficient power extraction.

The inclusion of $CSI$ as a second input variable enhances the algorithm’s ability to dynamically adjust the duty cycle step size based on the rate of change of SInC, enabling faster convergence and improved accuracy near the MPP. This makes the algorithm particularly effective under rapidly changing environmental conditions.

However, the algorithm’s performance remains sensitive to the precision of measurement instruments, as accurate calculations of $SInC$ and $CSI$ are critical for optimal operation. Additionally, careful tuning of membership functions and fuzzy rules is essential to maximize the controller’s effectiveness across different irradiance levels. These design considerations ensure that the algorithm maintains high performance and adaptability in real-world applications.

Table 6 quantifies the simulation results by comparing the average MPPT efficiency, convergence time to the MPP, root mean square (RMS) and root mean square error (RMSE) for each method. For simplicity and readability, the algorithms are abbreviated as follows:

**Table 6:** Performances of the proposed MPPT algorithms.

MPPT algorithms	Average MPPT efficiency (%)	Convergence time	RMSE	RMS
P&O algorithm	95.41	Min: 30 ms Max: 125 ms Average: 65 ms	8.98	96.34%
InC algorithm	95.6	Min: 30 ms Max: 115 ms Average: 60 ms	8.9	96.4
Algorithm 1	97.5	Min: 35 ms Max: 104 ms Average: 56.6 ms	8.66	97.65%
Algorithm 2	96.82	Min: 35 ms Max: 127 ms Average: 70.87 ms	9.1	97.18%
Algorithm 3	96.6	Min: 30 ms Max: 135 ms Average: 76 ms	9	96.89%
Proposed algorithm	97.7	Min: 26 ms Max: 89 ms Average: 53.5 ms	8.6	97.8%

- *Algorithm 1*: The first Hybrid P&O-FLC algorithm (using $\Delta P/\Delta V{ }$ and $\Delta P$ as input variables).
- *Algorithm 2*: The second Hybrid P&O-FLC algorithm (using $\Delta P/\Delta V{ }$ and its rate of change ($CE$) as input variables).
- *Algorithm 3*: The third Hybrid InC-FLC algorithm (using $SInC$ as the input variable).
- *Proposed Algorithm*: The fourth Hybrid InC-FLC algorithm (using $SInC$ and $CSI$ as input variables).

The proposed algorithm achieves the highest average MPPT efficiency (97.7%), the fastest average convergence time (53.5 ms), the lowest RMSE (8.6), and the highest RSM (97.8%), outperforming both conventional and other hybrid FLC methods. These results highlight the robustness and adaptability of the proposed algorithm, making it a promising solution for real-world PV systems operating in dynamic and unpredictable environments.

### Testing robustness under dynamic load variation

**Fig. 23:**
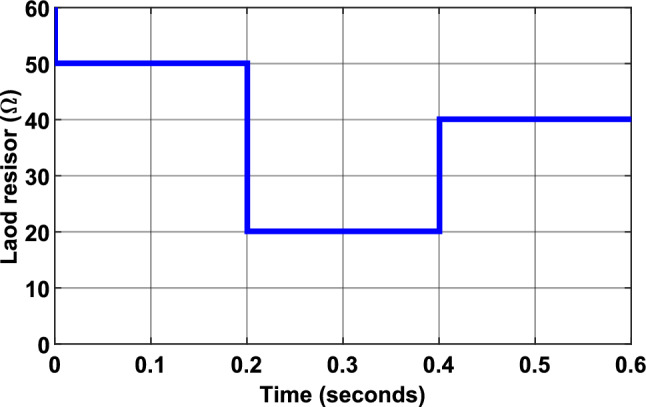
Load variation profile.

**Fig. 24:**
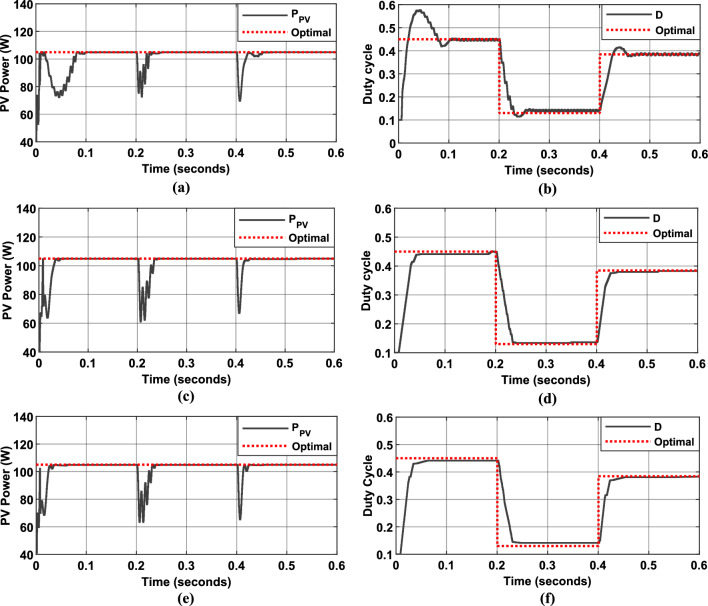
Simulation results of PV System under load variation and under constant irradiance of $500$ W/m^2^: (**a**) PV Power output, (**b**) duty cycle of PWM signal using P&O MPPT algorithm. (**c**) PV Power output, (**d**) duty cycle of PWM signal using the first Hybrid MPPT controller. (**e**) PV Power output, (**f**) duty cycle of PWM signal using the proposed Hybrid MPPT controller.

**Fig. 25:**
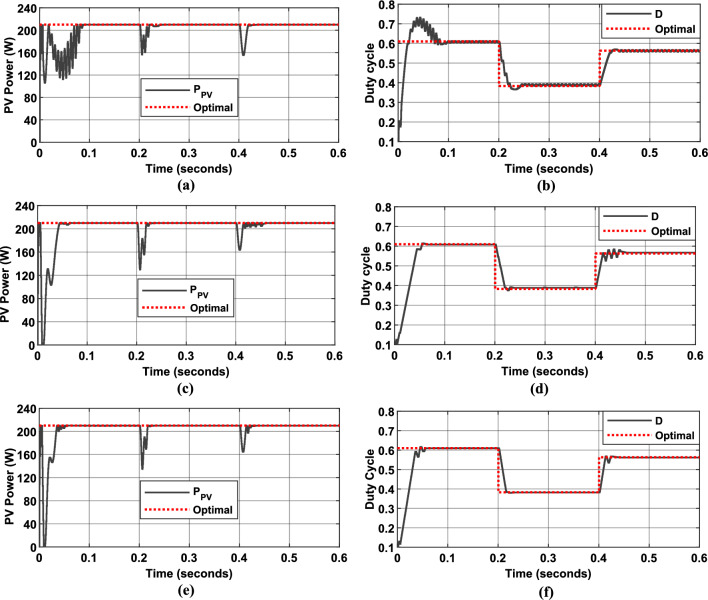
Simulation results of PV System under load variation and under constant irradiance of $1000$ W/m^2^: (**a**) PV Power output, (**b**) duty cycle of PWM signal using P&O MPPT algorithm. (**c**) PV Power output, (**d**) duty cycle of PWM signal using the first Hybrid MPPT controller. (**e**) PV Power output, (**f**) duty cycle of PWM signal using the proposed Hybrid MPPT controller.

To provide a more comprehensive evaluation of the proposed MPPT control strategy’s robustness, as well as that of the other algorithms under study, simulations were performed under conditions of dynamic load variation. As illustrated in Fig. 23, the load resistance was abruptly changed from $50 {\Omega }$ to $20 {\Omega }$ at $t = 0.2s$, and then from $20 \Omega$ to $35 \Omega$ at $t = 0.4s$. These tests were performed under two different irradiance levels: $500$ W/m^2^ (Fig. 24) and $1000$ W/m^2^ (Fig. 25).

Figures 24 and 25 illustrate the simulated behavior of a PV system using three different MPPT algorithms:

- *Figures a and b* Results obtained with the Incremental Conductance (InC) MPPT algorithm.
- *Figures c and d* Results from the first Hybrid FLC MPPT algorithm (using $\Delta P/\Delta V$ and $\Delta P$ as input variables).
- *Figures e and f* Results achieved with the proposed Hybrid FLC MPPT algorithm (using $SInC$ and its rate of change ($CSI$) as fuzzy input variables).

The figures illustrate the PV panel power and the duty cycle evolution, along with their corresponding optimal values (represented by dotted red lines) over the simulation period. These results highlight the consistent performance of the PV system and the proposed MPPT control strategy, even under sudden load variations.

Table 7 compares the performance of the proposed Hybrid InC-FLC algorithm with the traditional Incremental Conductance (InC) algorithm and the first P&O Hybrid FLC approach under two irradiance levels (1000 W/m^2^ and 500 W/m^2^) under load variation. The metrics used for evaluation are the RMSE and the RSM, which measure tracking accuracy and system efficiency, respectively.

**Table 7:** Performances of the proposed MPPT algorithms.

Irradiance level	MPPT algorithms	RMSE	RMS (%)
$1000$ W/m^2^	InC algorithm	27.11	97.14
	Algorithm 1	27.4690	96.94
	Proposed algorithm	25.1514	97.92
$500$ W/m^2^	InC algorithm	10.3091	96.57
	Algorithm 1	10.2597	97.35
	Proposed algorithm	9.3007	97.88

The proposed Hybrid InC-FLC algorithm demonstrated superior performance under dynamic load variations, achieving the lowest RMSE (25.15 at 1000 W/m^2^ and 9.3 at 500 W/m^2^) and the highest RSM (97.92% at 1000 W/m^2^ and 97.88% at 500 W/m^2^) compared to both the Incremental Conductance (InC) algorithm and the first Hybrid FLC algorithm. These results confirm the algorithm’s ability to maintain high accuracy, fast convergence, and stability even under sudden load changes and varying irradiance levels. While the InC and first Hybrid FLC algorithms also performed well, the proposed algorithm consistently showed lower oscillations and better adaptability, making it a robust and reliable solution for real-world PV systems operating in dynamic environments.

Table 8 provides a comprehensive overview of the algorithms’ performance, detailing their respective advantages and disadvantages. For instance, the P&O algorithm is easy to implement and requires minimal computational resources but may exhibit oscillations around the MPP and can be slow to converge under rapidly changing conditions. The InC algorithm shows fewer oscillations and faster convergence compared to P&O but remains sensitive to measurement accuracy. The hybrid FLC algorithms (Algorithm 1, Algorithm 2, and Algorithm 3) demonstrate improved performance, with better tracking accuracy and reduced oscillations. However, they face challenges such as constant P–V slopes to the left of the MPP or sensitivity to measurement precision. The proposed algorithm stands out for its excellent MPP tracking performance under various conditions, achieving higher overall efficiency and eliminating steady-state oscillations. However, it remains sensitive to measurement accuracy and division calculations, which are critical for optimal performance.

**Table 8:** Advantage and disadvantage of MPPT algorithms.

MPPT algorithms	Advantages	Disadvantages
P&O algorithm	Presents a practical implementation It requires minimal computational resources	Can experience fluctuations in electrical output around the MPP during steady-state The choice of step size can influence the algorithm’s performance It may be slow to converge to the MPP under rapidly changing conditions
InC algorithm	It exhibits fewer oscillations around the MPP It generally converges to the MPP more quickly than the P&O algorithm It can achieve higher precision in tracking the MPP	It may present oscillations during steady-state operation Its performance can be affected by the accuracy of measurement instruments
Algorithm 1	Efficiently establishes the operating point’s location concerning the MPP Demonstrates good MPP tracking performance on both sides of the MPP using $\Delta P.$ $\Delta P$ as a second input can enhance MPPT performance under low irradiance conditions Well-implemented fuzzy control can eliminate steady-state oscillations Can achieve higher overall MPPT performance	The P–V slope ($\Delta P/\Delta V$ ) can become constant to the left of the MPP, hindering the algorithm’s performance
Algorithm 2	Accurately locates the operating point relative to the MPP Demonstrates good MPP tracking performance on the right side of the MPP The second input CE can be used to minimize oscillations around the MPP during steady-state operation	$\Delta P/\Delta V$ can become constant to the left of the MPP, hindering the algorithm’s performance Precision around the MPP may be lower under low irradiance conditions While oscillations can be reduced, they may still persist under certain conditions, especially if the controller is optimized for a wide range of operating conditions
Algorithm 3	It is more efficient and easier to implement compared to previous algorithms The use of a single input variable (SInC) simplifies the control process Accurately locates the operating point relative to the MPP Demonstrates good MPP tracking performance on both sides of the MPP	Its performance can be affected by the accuracy of measurement instruments Can experience variations in electrical output around the MPP under steady-state conditions Issue division calculations
Proposed algorithm	Easily identifies the position of the operating point relative to the MPP Demonstrates excellent MPP tracking performance on both sides of the MPP under various conditions Can achieve higher overall MPPT performance Well-implemented fuzzy control can eliminate steady-state oscillations	Its performance can be affected by the accuracy of measurement instruments Issue division calculations

## Conclusion

This study evaluates conventional and hybrid fuzzy logic control (FLC) methods for MPPT in PV systems, demonstrating the superiority of hybrid FLC techniques under dynamic conditions. Conventional methods like P&O and InC achieve approximately 95% MPPT efficiency but suffer from oscillations and slow convergence under rapidly changing irradiance. Among the hybrid methods, the proposed approach using $SInC$ and its rate of change ($CSI$) as fuzzy inputs achieves the best performance, with an average efficiency of 97.7%, a convergence time of 53 ms, and a RMS of 97.8%. This method maintains stable electrical quantities across varying irradiance levels and under load variations, effectively eliminating oscillations and ensuring reliable operation under dynamic conditions.

While the first Hybrid-FLC method, using $\Delta P/\Delta V$ and $\Delta P$ as input variables, achieves 97.5% efficiency, it exhibits slight overshoots during abrupt irradiance changes. The second method ($\Delta P/\Delta V$ and $CE$) improves convergence speed under low irradiance but introduces minor oscillations under high irradiance. The third method ($SInC$ only) simplifies design but is sensitive to measurement precision and oscillations.

Interestingly, during simulations, we observed that the proposed method occasionally exhibited a slight delay (less than 5 ms) in responding to sudden load variations. While this delay did not significantly impact overall efficiency or stability, it highlights the importance of further refining the algorithm’s responsiveness to abrupt changes in operating conditions. These findings, combined with the low RMSE and high RMS, underscore the robustness and adaptability of the proposed method, making it a promising solution for real-world PV systems operating in dynamic environments.

In conclusion, the choice of input variables and fuzzy rule base design significantly impacts MPPT performance. The proposed hybrid FLC methods offer a robust and efficient solution for PV systems operating under dynamic conditions, making them highly suitable for real-world applications.

## Data Availability

The datasets used and/or analysed during the current study is available from Prof. Mustapha Melhaoui (m.melhaoui@uca.ma).

## Abbreviations

CE: Changing error
$CSI$: Rate of change of $SInC$
$$D$$: Duty cycle
FLC: Fuzzy logic control
InC: Incremental conductance
MPPT: Maximum power point tracking
P&O: Perturbation and observation
PWM: Pulse width modulation
RMS: Root mean square
RMSE: Root mean square error
$SInC$: Sum of conductance and incremental conductance
STC: Standard test condition
$C_{PV}$: Conductance of PV panel
$P_{PV}$: Power of PV module
$R_{mpp}$: Optimal resistance of PV panel
$V_{PV}$: PV panel’s voltage
$I_{PV}$: PV panel’s current
$$\Delta V$$: Changing of $$V_{PV}$$
$$\Delta P$$: Changing of $$P_{PV}$$
$\Delta D$: Duty cycle increment

## References

[1] Tomabechi, K. Energy resources in the future. *Energies* **3**, 686–695. 10.3390/en3040686 (2010).

[2] Lin, X. M. et al. A multi-criteria framework for designing of stand-alone and grid-connected photovoltaic, wind, battery clean energy system considering reliability and economic assessment. *Energy* **224**, 120154. 10.1016/j.energy.2021.120154 (2021).

[3] Guo, L., Meng, Z., Sun, Y. & Wang, L. A modified cat swarm optimization based maximum power point tracking method for photovoltaic system under partially shaded condition. *Energy* **144**, 501–514. 10.1016/j.energy.2017.12.059 (2018).

[4] Hassan, S. Z. et al. Neuro-fuzzy wavelet based adaptive MPPT algorithm for photovoltaic systems. *Energies* **10**, 394. 10.3390/en10030394.P (2017).

[5] Bamisile, O., Acen, C., Cai, D., Huang, Q. & Staffell, I. The environmental factors affecting solar photovoltaic output. *Renew. Sustain. Energy Rev.* **208**, 115073. 10.1016/j.rser.2024.115073 (2025).

[6] Bhatnagar, P. & Nema, R. K. Maximum power point tracking control techniques: State-of-the-art in photovoltaic applications. *Renew. Sustain. Energy Rev* **23**, 224–241. 10.1016/j.rser.2013.02.011 (2013).

[7] Silva, I. F., Vicente, P. D. S., Tofoli, F. L. & Vicente, E. M. Plotting characteristic curves of photovoltaic modules: A simple and portable approach. *IEEE Ind. Appl. Mag* **27**(3), 63–72 (2021).

[8] Padmanaban, S. et al. A hybrid photovoltaic-fuel cell for grid integration with Jaya-based maximum power point tracking: Experimental performance evaluation. *IEEE Access* **7**, 82978–82990 (2019).

[9] Singh, D. & RiaYadav, J. Perturb and observe method MATLAB simulink and design of PV system using buck boost converter. *Int. J. Sci. Eng. Technol. Res.* **3**, 1692–1695 (2014).

[10] Kumar, N., Hussain, I., Singh, B. & Panigrahi, B. K. Framework of maximum power extraction from solar PV panel using self predictive perturb and observe algorithm. *IEEE Trans. Sustain. Energy* **9**(2), 895–903 (2018).

[11] Khan, O., Acharya, S., Hosani, M. A. & Moursi, M. S. E. Hill climbing power flow algorithm for hybrid DC/AC microgrids. *IEEE Trans. Power Electron* **33**(7), 5532–5537 (2018).

[12] Jately, V., Azzopardi, B., Joshi, J., Sharma, A. & Arora, S. Experimental analysis of hill-climbing MPPT algorithms under low irradiance levels. *Renew. Sustain. Energy Rev.* **150**, 111467. 10.1016/j.rser.2021.111467 (2021).

[13] Saravana, S. D. Modeling and simulation of incremental conductance MPPT algorithm for photovoltaic applications. *Int. J. Sci. Eng. Technol.* **2**, 681–685 (2013).

[14] Sivakumar, P., Kader, A. A., Kaliavaradhan, Y. & Arutchelvi, M. Analysis and enhancement of PV efficiency with incremental conductance MPPT technique under non-linear loading conditions. *Renew. Energy* **81**, 543–550. 10.1016/j.renene.2015.03.062 (2015).

[15] Loukriz, A., Haddadi, M. & Messalti, S. Simulation and experimental design of a new advanced variable step size incremental conductance MPPT algorithm for PV systems. *ISA Trans.* **62**, 30–38. 10.1016/j.isatra.2015.08.006 (2016). 26337741 10.1016/j.isatra.2015.08.006

[16] Boukenoui, R., Salhi, H., Bradai, R. & Mellit, A. A new intelligent MPPT method for stand-alone photovoltaic systems operating under fast transient variations of shading patterns. *Sol. Energy* **124**, 124–142. 10.1016/j.solener.2015.11.023 (2016).

[17] Saady, I. et al. Improving photovoltaic water pumping system performance with ANN-based direct torque control using real-time simulation. *Sci. Rep.* **15**(1), 4024. 10.1038/s41598-025-88330-8 (2025). 39894812 10.1038/s41598-025-88330-8 PMC11788436

[18] Djilali, A. B. et al. Enhanced variable step sizes perturb and observe MPPT control to reduce energy loss in photovoltaic systems. *Sci. Rep.* **15**, 11700. 10.1038/s41598-025-95309-y (2025). 40188188 10.1038/s41598-025-95309-y PMC11972299

[19] Priyadarshi, N., Padmanaban, S., Holm-Nielsen, J. B., Blaabjerg, F. & Bhaskar, M. S. An experimental estimation of hybrid ANFIS–PSO-based MPPT for PV grid integration under fluctuating sun irradiance. *IEEE Syst. J.* **14**(1), 1218–1229. 10.1109/JSYST.2019.2949083 (2020).

[20] Jabbar, R. I., Mekhilef, S., Mubin, M., Alshammari, O. & Kazaili, A. A fast MPPT method based on improved water cycle optimization algorithm for photovoltaic systems under partial shading conditions and load variations. *IEEE Open J. Ind. Electron. Soc.* **5**, 1324–1338. 10.1109/OJIES.2024.3510367 (2024).

[21] Alshareef, M. J. An effective falcon optimization algorithm based MPPT under partial shaded photovoltaic systems. *IEEE Access* **10**, 131345–131360. 10.1109/ACCESS.2022.3226654 (2022).

[22] Bollipo, R. B., Mikkili, S. & Bonthagorla, P. K. Hybrid, optimal, intelligent and classical PV MPPT techniques: A review. *CSEE J. Power Energy Syst.* **7**(1), 9–33. 10.17775/CSEEJPES.2019.02720 (2020).

[23] Khan, M. J., Mathew, L., Alotaibi, M. A., Malik, H. & Nassar, M. E. Fuzzy-logic-based comparative analysis of different maximum power point tracking controllers for hybrid renewal energy systems. *Mathematics* **10**, 529. 10.3390/math10030529 (2022).

[24] Ali, Z. M. et al. Novel hybrid improved bat algorithm and fuzzy system based MPPT for photovoltaic under variable atmospheric conditions. *Sustain. Energy Technol. Assess.* **52**, 102156. 10.1016/j.seta.2022.102156 (2022).

[25] Ou, T.-C., Su, W.-F., Liu, X.-Z., Huang, S.-J. & Tai, T.-Y. A modified bird-mating optimization with hill-climbing for connection decisions of transformers. *Energies* **9**, 671. 10.3390/en9090671 (2016).

[26] Robles Algarín, C., Taborda Giraldo, J. & Rodríguez Álvarez, O. Fuzzy logic based MPPT controller for a PV system. *Energies* **10**, 2036. 10.3390/en10122036 (2017).

[27] Chen, L., Amirahmadi, A., Zhang, Q., Kutkut, N. & Batarseh, I. Design and implementation of three-phase two-stage grid-connected module integrated converter. *IEEE Trans. Power Electron.* **29**(8), 3881–3892. 10.1109/TPEL.2013.2294933 (2014).

[28] NM, J. S. MPPT of solar PV systems using PSO memetic algorithm considering the effect of change in tilt angle. *Sci. Rep.* **15**(1), 1–17 (2025). 40050374 10.1038/s41598-025-92598-1 PMC11885664

[29] Belghiti, H. et al. A novel adaptive FOCV algorithm with robust IMRAC control for sustainable and high-efficiency MPPT in standalone PV systems: Experimental validation and performance assessment. *Sci. Rep.* **14**, 31962. 10.1038/s41598-024-83512-2 (2024). 39738769 10.1038/s41598-024-83512-2 PMC11685883

[30] Dutu, L. C., Mauris, G. & Bolon, P. A. Fast and Accurate rule-base generation method for Mamdani fuzzy systems. *IEEE Trans. Fuzzy Syst* **26**, 715–733 (2018).

[31] Shiau, J.-K., Wei, Y.-C. & Chen, B.-C. A study on the fuzzy-logic-based solar power MPPT algorithms using different fuzzy input variables. *Algorithms* **8**, 100–127. 10.3390/a8020100 (2015).

[32] Kim, J.-C., Huh, J.-H. & Ko, J.-S. Optimization design and test bed of fuzzy control rule base for PV system MPPT in micro grid. *Sustainability* **12**(9), 3763. 10.3390/su12093763 (2020).

[33] Ullah, K. et al. Maximum power point technique (MPPT) for PV system based on improved pert and observe (P&O) method with PI controller. *Int. Res. J. Eng. Technol. (IRJET)* **6**(12), 813–819 (2019).

[34] Yilmaz, U., Kircay, A. & Borekci, S. PV system fuzzy logic MPPT method and PI control as a charge controller. *Renew. Sustain. Energy Rev.* **81**(P1), 994–1001. 10.1016/j.rser.2017.08.048 (2018).

[35] Nataraj, C. et al. Comparative analysis of direct coupling and MPPT control in standalone PV systems for solar energy optimization to meet sustainable building energy demands. *Sci. Rep.* **14**, 22924. 10.1038/s41598-024-72606-6 (2024). 39358408 10.1038/s41598-024-72606-6 PMC11446928

[36] Zemmit, A. et al. GWO and WOA variable step MPPT algorithms-based PV system output power optimization. *Sci. Rep.* **15**, 7810. 10.1038/s41598-025-89898-x (2025). 40050315 10.1038/s41598-025-89898-x PMC11885651

[37] Wandhare, R. G. & Agarwal, V. Novel integration of a PV-wind energy system with enhanced efficiency. *IEEE Trans. Power Electron.* **30**(7), 3638–3649. 10.1109/TPEL.2014.2345766 (2015).

[38] Mishra, S. & Achary, B. S. A novel controller for a grid connected single phase PV system and its real time implementation. in *2014 IEEE PES General Meeting | Conference & Exposition, National Harbor, MD, USA* 1–5 (2014). 10.1109/PESGM.2014.6939836

[39] Hayat, A. et al. Design and analysis of input capacitor in DC–DC boost converter for photovoltaic-based systems. *Sustainability* **15**(7), 6321. 10.3390/su15076321 (2023).

[40] Ying, H. Conditions for general Mamdani fuzzy controllers to be nonlinear. in *2002 Annual Meeting of the North American Fuzzy Information Processing Society Proceedings. NAFIPS-FLINT 2002 (Cat. No. 02TH8622)* 201–203.

[41] Mamdani, E. H. & Assilian, S. An experiment in linguistic synthesis with a fuzzy logic controller. *Int. J. Man-Mach. Stud.* **7**(1), 1–13 (1975).

